# Experimental data on the mechanical and thermal properties of extruded composites from recycled wind turbine blade material

**DOI:** 10.1016/j.dib.2019.104253

**Published:** 2019-07-15

**Authors:** Seyed Hossein Mamanpush, Azadeh Tavousi Tabatabaei, Hui Li, Karl Englund

**Affiliations:** Composite Materials and Engineering Center, Washington State University, Pullman, WA 99163, USA

**Keywords:** Recycling, Wind turbine blade, Polymer-matrix composite, Glass fiber

## Abstract

The wind turbine blades (WTB) that face end-of-life was first mechanically milled and classified through a range of varying screen sizes. We then blended this with high density polyethylene (HDPE) thermoplastic resin and extruded it to a profiled composite. We determined the influence of refined particle size, resin content and coupling agents (maleic anhydride polyethylene (MAPE) and methacryloxypropyltriethoxysilane (Silane)) on the mechanical and CLTE properties of recycled composites (Mamanpush et al., 2019).

**Table:** Specifications table

Subject area	Composite material
More specific subject area	*Recycling thermoset composite material*
Type of data	*Table*
How data was acquired	*Mechanical properties are obtained by experimental method using ASTM D790-17.* *CLTE properties are obtained by experimental method using ASTM D1037-12.*
Data format	*Raw, analyzed*
Experimental factors	*Mechanical and thermal properties of composite material*
Experimental features	*All composite specimens were undergone flexural and CLTE test in dry condition*
Data source location	*Composites Materials and Engineering Center at Washington State University*
Data accessibility	*Data is with the article*
Related research article	*Seyed Hossein Mamanpush, Hui Li, Karl Englund, Azadeh Tavousi Tabatabaei . Extruded fiber-reinforced composites manufactured from recycled wind turbine blade material. Waste and biomass valorization, 1–10.**https://doi.org/10.1007/s12649-019-00659-0**[1]**.*

**Table:** 

**Value of the data** • Fibers and other fillers significantly reduce thermal expansion. The degree of anisotropy of the filler and the filler orientation pose great impact on the linear coefficient of thermal expansion, therefore, characterizing CLTE is vital for fiber-reinforced composites. • CLTE helps determine dimensional behavior of structures subject to temperature changes. • Presented dataset shows consistency among the samples and helps researcher to see the actual trend among these second-generation composites with different formulation. • Raw dataset presented on mechanical properties of rWTB composites helps other researcher in this field to understand the original condition of these second-generation composites. • Data on Mechanical properties of second-generation composites manufactured from rWTB materials gives the researchers clear vision about the potential utilization of these second-generation composites.

## 1 Data

For obtaining mechanical properties of thermoplastic composites fabricated using recycled wind turbine blade materials, flexural tests were performed based on ASTM D790-17. We determined the influence of refined particle size, resin content and coupling agents (maleic anhydride polyethylene (MAPE) and methacryloxypropyltriethoxysilane (Silane)) on the mechanical properties of recycled composites (Table 3).

**Table 1:** Extruded rWTB composite formulation.

Sample #	Pine (40 mesh) (%)	Pine (60 mech) (%)	3.18 mm MSS rWTB(%)	1.59 mm MSS rWTB (%)	Talc (%)	MAP (%)	Silane (%)	HDPE (%)
1	55		0		6			36
2			40		6			51
3			45		6			46
4			50		6			41
5			55		6			36
6			60		6			31
7			50					47
8			55					42
9			60					37
10		55						42
11		55			6			36
12		55				2		40
13		54.5					0.5	42
14				50				47
15				55				42
16				60				37
17				50		2		45
18				55		2		40
19				60		2		35
20				49.5			0.5	47
21				54.5			0.5	42
22				59.5			0.5	37
23		13.75		41.25				42
24		27.5		27.5				42
25		41.25		13.75				42
26				65		2		30
27				70		2		25
28				65				32
29				70				27

**Table 2:** Physical properties of each specimen prepared for Flexural test.

Sample #	Depth (In)			Width (In)			Average (In)	
							Depth	Width
1–1	0.3815	0.385	0.3885	1.498	1.486	1.489	0.385	1.491
1–2	0.378	0.371	0.373	1.4905	1.4755	1.4835	0.374	1.483
1–3	0.373	0.369	0.371	1.483	1.4815	1.4815	0.371	1.482
1–4	0.376	0.375	0.3735	1.484	1.487	1.485	0.374	1.485
1–5	0.3735	0.374	0.3765	1.4815	1.4815	1.4855	0.374	1.482
1–6	0.374	0.369	0.3715	1.488	1.482	1.4855	0.371	1.485
3–1	0.45	0.457	0.442	1.5075	1.5185	1.523	0.449	1.516
3–2	0.452	0.465	0.463	1.502	1.5415	1.531	0.460	1.524
3–3	0.433	0.434	0.431	1.505	1.501	1.498	0.432	1.501
3–4	0.423	0.428	0.4295	1.48	1.4885	1.503	0.426	1.490
3–5	0.44	0.4555	0.4495	1.492	1.5085	1.517	0.448	1.505
3–6	0.4255	0.427	0.434	1.492	1.485	1.489	0.428	1.488
4–1	0.4385	0.428	0.4235	1.5095	1.5005	1.499	0.43	1.503
4–2	0.423	0.4265	0.4255	1.49	1.498	1.5105	0.425	1.4995
4–3	0.442	0.445	0.4515	1.5145	1.5065	1.517	0.446	1.512
4–4	0.4285	0.4195	0.4225	1.5005	1.4965	1.4915	0.423	1.496
4–5	0.434	0.4365	0.4375	1.512	1.5125	1.5105	0.436	1.511
4–6	0.4375	0.437	0.4475	1.5025	1.51	1.5125	0.440	1.508
5–1	0.3965	0.395	0.3955	1.4815	1.48	1.481	0.395	1.480
5–2	0.3985	0.391	0.3935	1.4755	1.476	1.4785	0.394	1.476
5–3	0.3995	0.39	0.388	1.478	1.4795	1.4745	0.392	1.477
5–4	0.4	0.4005	0.3955	1.491	1.4815	1.4865	0.398	1.486
5–5	0.401	0.398	0.3955	1.492	1.4845	1.479	0.398	1.485
5–6	0.398	0.3955	0.3935	1.482	1.4705	1.471	0.395	1.474
6–1	0.3875	0.3885	0.389	1.4855	1.4805	1.481	0.388	1.482
6–2	0.389	0.3935	0.392	1.4985	1.4865	1.479	0.391	1.488
6–3	0.392	0.387	0.387	1.494	1.4805	1.4825	0.388	1.485
6–4	0.391	0.389	0.387	1.477	1.4725	1.4725	0.389	1.474
6–5	0.3915	0.392	0.389	1.4755	1.4755	1.469	0.390	1.473
6–6	0.391	0.3925	0.39	1.478	1.4815	1.484	0.391	1.481
7–1	0.412	0.414	0.4035	1.485	1.477	1.477	0.409	1.479
7–2	0.404	0.419	0.4115	1.4765	1.464	1.475	0.411	1.471
7–3	0.411	0.4085	0.4145	1.473	1.471	1.4815	0.411	1.475
7–4	0.419	0.418	0.408	1.481	1.482	1.4845	0.415	1.482
7–5	0.399	0.4145	0.412	1.486	1.4705	1.4765	0.408	1.477
7–6	0.414	0.415	0.418	1.4945	1.4855	1.4955	0.415	1.491
8–1	0.399	0.41	0.402	1.479	1.482	1.4825	0.401	1.481
8–2	0.3995	0.4055	0.406	1.466	1.467	1.467	0.403	1.466
8–3	0.4	0.4015	0.3965	1.4995	1.481	1.483	0.399	1.487
8–4	0.4035	0.406	0.399	1.483	1.469	1.4525	0.402	1.468
8–5	0.399	0.398	0.3995	1.4715	1.4685	1.473	0.398	1.471
8–6	0.4015	0.396	0.403	1.493	1.4845	1.4885	0.400	1.488
9–1	0.396	0.3985	0.398	1.4995	1.4875	1.485	0.397	1.490
9–2	0.397	0.393	0.3935	1.492	1.491	1.494	0.394	1.492
9–3	0.403	0.3965	0.394	1.487	1.489	1.484	0.397	1.486
9–4	0.405	0.393	0.394	1.502	1.478	1.4795	0.397	1.486
9–5	0.412	0.4065	0.408	1.497	1.4905	1.502	0.408	1.496
9–6	0.401	0.3895	0.4	1.4935	1.462	1.4605	0.396	1.472
10–1	0.385	0.389	0.3855	1.479	1.477	1.478	0.386	1.478
10–2	0.389	0.3845	0.388	1.4835	1.478	1.473	0.387	1.478
10–3	0.395	0.3865	0.386	1.479	1.4785	1.4745	0.389	1.477
10–4	0.396	0.3885	0.3845	1.4755	1.4725	1.474	0.389	1.474
10–5	0.387	0.3865	0.391	1.485	1.48	1.474	0.388	1.479
10–6	0.387	0.39	0.3905	1.4745	1.4745	1.4745	0.389	1.474
11–1	0.39	0.382	0.384	1.483	1.4815	1.481	0.386	1.481
11–2	0.395	0.3835	0.3845	1.4805	1.48	1.48	0.387	1.480
11–3	0.392	0.381	0.385	1.49	1.481	1.483	0.386	1.484
11–4	0.394	0.382	0.383	1.483	1.481	1.479	0.389	1.481
11–5	0.3855	0.3825	0.3805	1.483	1.4805	1.479	0.382	1.480
11–6	0.385	0.3835	0.382	1.4825	1.485	1.431	0.383	1.466
12–1	0.3885	0.381	0.384	1.454	1.461	1.458	0.384	1.457
12–2	0.378	0.385	0.3895	1.4505	1.4405	1.444	0.384	1.445
12–3	0.385	0.38	0.399	1.4505	1.4505	1.444	0.388	1.448
12–4	0.3835	0.3795	0.3755	1.4745	1.473	1.47	0.379	1.472
12–5	0.3815	0.38	0.38	1.457	1.4705	1.46	0.380	1.462
12–6	0.376	0.38	0.378	1.464	1.4615	1.4625	0.378	1.46
14–1	0.424	0.442	0.4345	1.5105	1.502	1.4985	0.433	1.503
14–2	0.4385	0.436	0.437	1.504	1.501	1.51	0.4371	1.505
14–3	0.435	0.431	0.4295	1.4915	1.484	1.4835	0.431	1.486
14–4	0.438	0.443	0.438	1.4955	1.504	1.4995	0.439	1.499
14–5	0.4325	0.436	0.4395	1.5175	1.512	1.514	0.436	1.514
14–6	0.448	0.448	0.433	1.513	1.5015	1.5045	0.443	1.506
15–1	0.4185	0.4245	0.4275	1.479	1.4835	1.482	0.423	1.481
15–2	0.4335	0.4285	0.4215	1.47	1.475	1.475	0.429	1.473
15–3	0.437	0.445	0.446	1.5065	1.493	1.5055	0.444	1.501
15–4	0.425	0.4285	0.423	1.4925	1.4865	1.489	0.425	1.488
15–5	0.4235	0.4245	0.4315	1.4815	1.478	1.477	0.426	1.478
15–6	0.441	0.429	0.4285	1.4835	1.488	1.4735	0.4328	1.4816
16–1	0.4085	0.4085	0.406	1.482	1.4785	1.4805	0.407	1.488
16–2	0.408	0.4155	0.408	1.477	1.483	1.481	0.410	1.480
16–3	0.413	0.409	0.411	1.4845	1.4825	1.474	0.411	1.480
16–4	0.4085	0.412	0.408	1.481	1.471	1.469	0.409	1.473
16–5	0.409	0.4095	0.412	1.479	1.482	1.4755	0.410	1.479
16–6	0.4115	0.4085	0.417	1.483	1.483	1.4795	0.412	1.481
17–1	0.409	0.419	0.42	1.4705	1.471	1.4685	0.416	1.477
17–2	0.413	0.418	0.4095	1.4705	1.463	1.446	0.413	1.459
17–3	0.413	0.415	0.41	1.454	1.452	1.466	0.412	1.457
17–4	0.419	0.412	0.413	1.473	1.48	1.453	0.414	1.468
17–5	0.404	0.4045	0.4035	1.4705	1.463	1.472	0.404	1.468
17–6	0.42	0.4125	0.424	1.461	1.461	1.4605	0.418	1.460
18–1	0.404	0.404	0.406	1.454	1.464	1.451	0.404	1.45
18–2	0.402	0.401	0.403	1.444	1.4485	1.4455	0.402	1.446
18–3	0.4035	0.4065	0.4045	1.4505	1.4445	1.453	0.404	1.449
18–4	0.402	0.4045	0.391	1.46	1.4645	1.4425	0.399	1.455
18–5	0.397	0.3945	0.3925	1.457	1.455	1.4535	0.394	1.455
18–6	0.411	0.409	0.411	1.4695	1.462	1.4655	0.410	1.465
19–1	0.389	0.385	0.3885	1.4455	1.449	1.441	0.387	1.445
19–2	0.384	0.385	0.388	1.452	1.457	1.456	0.385	1.455
19–3	0.394	0.39	0.392	1.46	1.463	1.461	0.392	1.461
19–4	0.392	0.392	0.3865	1.46	1.468	1.468	0.390	1.465
19–5	0.391	0.3885	0.3865	1.4615	1.4505	1.448	0.388	1.453
19–6	0.395	0.3935	0.391	1.4675	1.466	1.466	0.393	1.466
13–1	0.3765	0.375	0.378	1.485	1.482	1.4825	0.376	1.483
13–2	0.38	0.379	0.378	1.487	1.4835	1.4805	0.379	1.483
13–3	0.3805	0.379	0.3805	1.485	1.485	1.483	0.388	1.484
13–4	0.387	0.387	0.383	1.491	1.488	1.4795	0.385	1.486
13–5	0.3885	0.387	0.391	1.485	1.486	1.4815	0.388	1.484
13–6	0.387	0.3945	0.391	1.4815	1.488	1.483	0.390	1.489
20–1	0.4565	0.4445	0.437	1.5105	1.501	1.5005	0.446	1.504
20–2	0.436	0.454	0.4395	1.4985	1.4945	1.497	0.443	1.496
20–3	0.447	0.446	0.434	1.489	1.49	1.494	0.442	1.491
20–4	0.4525	0.446	0.452	1.4935	1.489	1.503	0.450	1.495
20–5	0.4675	0.446	0.431	1.502	1.5015	1.506	0.448	1.506
20–6	0.45	0.445	0.4515	1.499	1.5065	1.504	0.448	1.503
21–1	0.442	0.4185	0.4205	1.4785	1.473	1.4735	0.427	1.475
21–2	0.4145	0.4245	0.4005	1.477	1.48	1.477	0.413	1.478
21–3	0.405	0.4085	0.411	1.481	1.477	1.4785	0.408	1.478
21–4	0.409	0.412	0.4205	1.481	1.4785	1.4795	0.413	1.479
21–5	0.4135	0.409	0.404	1.485	1.4805	1.474	0.408	1.4798
21–6	0.3955	0.431	0.41	1.478	1.481	1.48	0.412	1.4796
22–1	0.3945	0.3935	0.389	1.4775	1.476	1.4785	0.392	1.477
22–2	0.392	0.389	0.3835	1.479	1.4875	1.479	0.388	1.481
22–3	0.394	0.39	0.396	1.4805	1.4755	1.477	0.393	1.477
22–4	0.392	0.3825	0.381	1.4835	1.475	1.484	0.385	1.480
22–5	0.3815	0.391	0.388	1.477	1.4755	1.48	0.386	1.477
22–6	0.39	0.3905	0.3985	1.4775	1.4745	1.479	0.393	1.477
23–1	0.393	0.393	0.387	1.4755	1.473	1.473	0.391	1.473
23–2	0.3925	0.3895	0.3935	1.474	1.471	1.4745	0.391	1.473
23–3	0.3895	0.389	0.387	1.479	1.4765	1.4775	0.388	1.477
23–4	0.391	0.388	0.388	1.4825	1.4765	1.482	0.389	1.480
23–5	0.392	0.3875	0.392	1.48	1.478	1.482	0.3905	1.48
23–6	0.3865	0.392	0.3925	1.476	1.4775	1.481	0.390	1.478
24–1	0.389	0.385	0.3855	1.477	1.4755	1.4745	0.386	1.475
24–2	0.385	0.386	0.3845	1.474	1.4745	1.476	0.385	1.474
24–3	0.383	0.3855	0.3845	1.481	1.4775	1.479	0.384	1.479
24–4	0.386	0.386	0.386	1.4815	1.476	1.476	0.386	1.477
24–5	0.38	0.387	0.387	1.472	1.472	1.474	0.384	1.472
24–6	0.386	0.3845	0.385	1.477	1.4735	1.472	0.385	1.474
25–1	0.386	0.386	0.3845	1.477	1.4765	1.4775	0.385	1.477
25–2	0.3835	0.3825	0.3815	1.482	1.48	1.479	0.382	1.480
25–3	0.3805	0.38	0.3825	1.4825	1.489	1.4795	0.381	1.483
25–4	0.382	0.386	0.383	1.481	1.479	1.481	0.383	1.480
25–5	0.386	0.3785	0.386	1.4805	1.4725	1.4695	0.383	1.487
25–6	0.39	0.391	0.3875	1.48	1.4745	1.477	0.389	1.477
26–1	0.373	0.375	0.3805	1.4515	1.4595	1.4555	0.376	1.455
26–2	0.379	0.3825	0.3805	1.4665	1.46	1.456	0.380	1.460
26–3	0.3835	0.384	0.383	1.461	1.4625	1.466	0.3835	1.463
26–4	0.3765	0.3785	0.384	1.451	1.448	1.449	0.379	1.448
26–5	0.3765	0.381	0.379	1.458	1.4635	1.4545	0.378	1.458
26–6	0.379	0.382	0.387	1.462	1.461	1.4605	0.382	1.461
27–1	0.3695	0.3735	0.3675	1.4515	1.452	1.446	0.370	1.447
27–2	0.379	0.3655	0.37	1.4555	1.4525	1.4515	0.371	1.453
27–3	0.3735	0.37	0.3675	1.454	1.4565	1.453	0.370	1.454
27–4	0.3665	0.377	0.3715	1.4535	1.453	1.454	0.371	1.453
27–5	0.37	0.3675	0.3665	1.464	1.457	1.458	0.368	1.457
27–6	0.3695	0.3735	0.3745	1.465	1.463	1.4625	0.372	1.463
28–1	0.3845	0.3875	0.3805	1.481	1.478	1.476	0.384	1.478
28–2	0.3905	0.383	0.3865	1.4815	1.483	1.488	0.386	1.484
28–3	0.383	0.3815	0.3765	1.4695	1.4745	1.4795	0.380	1.474
28–4	0.3835	0.3845	0.382	1.477	1.476	1.4755	0.383	1.47
28–5	0.3775	0.3815	0.379	1.477	1.481	1.473	0.379	1.477
28–6	0.376	0.377	0.373	1.476	1.4755	1.479	0.375	1.476
29–1	0.38	0.3775	0.376	1.4795	1.4795	1.4885	0.377	1.482
29–2	0.3855	0.383	0.382	1.4875	1.4885	1.482	0.383	1.486
29–3	0.375	0.377	0.38	1.4845	1.483	1.488	0.377	1.485
29–4	0.381	0.388	0.3875	1.486	1.4835	1.492	0.385	1.487
29–5	0.377	0.379	0.377	1.483	1.4815	1.48	0.379	1.481
29–6	0.39	0.3905	0.3985	1.4775	1.4745	1.479	0.393	1.477

**Table 3:** Flexural properties of rWTB composites.

Sample	Specimen	Density (lb/ft^3^)	MOE (Psi)	MOR (Psi)	Strain at break
**1**	1	66.5	304567.7	3231.804	0.0233567
	2	69.7	343208.1	3505.698	0.0236243
	3	70.3	330044.9	3380.67	0.0163448
	4	68.8	286116.8	3586.546	0.0215225
	5	69.5	281497.5	3327.194	0.0175193
	6	69.6	313466.8	3668.125	0.0194976
Average for Sample 1		69.07165	309817	3450.006	0.0203109
Standard Dev for Sample 1		1.349893	24202.09	165.4225	0.0030301
Coefficient of Variation for Sample 1		1.95%	7.81%	4.79%	14.92%
**3**	1	69.0	308541.1	3401.682	0.0276958
	2	68.5	306065.7	3452.762	0.0308106
	3	70.2	321223.7	3704.978	0.0319467
	4	70.2	305611.9	3647.846	0.0401398
	5	69.0	299410.5	3496.801	0.028492
	6	69.3	276882	3360.609	0.0408529
Average for Sample 3		69.39051	302955.8	3510.78	0.033323
Standard Dev for Sample 3		0.713198	14656.38	137.4883	0.0057685
Coefficient of Variation for Sample 3		1.03%	4.84%	3.92%	17.31%
**4**	1	72.8	362097	3800.943	0.0264318
	2	72.0	355692.3	3611.169	0.0226378
	3	71.0	349597.7	3825.238	0.0254042
	4	72.9	374619.8	3916.847	0.0231283
	5	72.3	350088.8	3741.625	0.0233838
	6	71.1	335608.3	3660.137	0.02385
Average for Sample 4		72.02719	354617.3	3759.326	0.0241393
Standard Dev for Sample 4		0.821483	13147.22	112.2421	0.0014688
Coefficient of Variation for Sample 4		1.14%	3.71%	2.99%	6.08%
**5**	1	77.1	407256.3	3920.144	0.0161263
	2	75.1	375962.4	3709.435	0.021672
	3	75.7	384892.7	3629.383	0.0159973
	4	76.3	375091.1	3749.674	0.0198154
	5	74.9	342725.6	3497.443	0.0200733
	6	75.1	357807.5	3544.593	0.0175873
Average for Sample 5		75.1	373955.9	3675.112	0.0185453
Standard Dev for Sample 5		0.847762	22225.86	153.278	0.0023232
Coefficient of Variation for Sample 5		1.13%	5.94%	4.17%	12.53%
**6**	1	80.2	429714.7	3863.973	0.0147796
	2	79.1	409733.6	3656.425	0.0141218
	3	79.2	382948.5	3559.357	0.0158962
	4	77.8	386033.9	3469.042	0.012982
	5	77.8	353509.5	3247.073	0.0133762
	6	78.5	377348	3531.565	0.0156096
Average for Sample 6		78.76046	389881.4	3554.573	0.0144609
Standard Dev for Sample 6		0.943874	26546.84	204.2186	0.0011798
Coefficient of Variation for Sample 6		1.20%	6.81%	5.75%	8.16%
**7**	1	70.6	291661.8	3519.779	0.0323017
	2	69.9	255131.4	3236.844	0.0362315
	3	70.3	282118.8	3609.505	0.0342891
	4	70.2	304974.3	3868.327	0.0386613
	5	69.8	318574.4	3746.874	0.0339369
	6	69.2	314786.3	3801.544	0.031994
Average For Sample 7		69.98004	294541.2	3630.479	0.0345691
Standard Dev For Sample 7		0.505225	23715.18	231.0952	0.0025203
Coefficient of Variation for Sample 7		0.72%	8.05%	6.37%	7.29%
**8**	1	71.1	324213.5	3483.48	0.024306
	2	70.7	311916.9	3375.537	0.0312424
	3	71.7	348652.7	3761.752	0.0264836
	4	70.8	300857.7	3348.814	0.0267157
	5	71.6	289232.5	3326.822	0.0253743
	6	71.2	342701.5	3669.565	0.0225609
Average for Sample 8		71.18565	319595.8	3494.328	0.0261138
Standard Dev for Sample 8		0.410923	23373.11	182.0357	0.0029407
Coefficient of Variation for Sample 8		0.58%	7.31%	5.21%	11.26%
**9**	1	73.0	362227.3	3569.507	0.0208864
	2	73.7	346902.1	3448.675	0.0205047
	3	72.9	332385.5	3397.423	0.0202824
	4	72.8	333415.3	3167.471	0.0203698
	5	73.1	336302.5	3367.432	0.0201465
	6	73.6	324021.2	3450.692	0.0211331
Average for Sample 9		73.1768	339209	3400.2	0.0205538
Standard Dev for Sample 9		0.40733	13477.89	133.273	0.00038
Coefficient of Variation for Sample 9		0.56%	3.97%	3.92%	1.85%
**10**	1	66.3	247309	3255	0.043
	2	66.6	227202	2759	0.034
	3	66.4	230768	2775	0.039
	4	66.1	232621	2971	0.041
	5	66.2	242181	3053	0.042
	6	66.4	247159	3036	0.038
Average for Sample 9		66.33112	237873.3	2974.788	0.0395191
Standard Dev for Sample 9		0.154142	8783.503	187.0119	0.0033784
Coefficient of Variation for Sample 9		0.23%	3.69%	6.29%	8.55%
**11**	1	69.7	268850	3083	0.033
	2	69.4	283206	2977	0.028
	3	69.5	252420	2937	0.033
	4	69.4	259069	2944	0.028
	5	68.8	291998	3358	0.029
	6	70.9	289147	3161	0.032
Average for Sample 9		69.59452	274114.9	3076.59	0.0302723
Standard Dev for Sample 9		0.687315	16451	163.2519	0.0024745
Coefficient of Variation for Sample 9		0.99%	6.00%	5.31%	8.17%
**12**	1	66.6	373882	4864	0.027
	2	67.3	350489	4618	0.030
	3	66.5	337832	4503	0.033
	4	68.2	353776	4135	0.025
	5	67.9	364422	4366	0.027
	6	68.2	377435	4386	0.027
Average for Sample 9		67.42186	359639.4	4478.469	0.0283046
Standard Dev for Sample 9		0.781465	15073.72	248.1113	0.0029043
Coefficient of Variation for Sample 9		1.16%	4.19%	5.54%	10.26%
**13**	1	68.0	253234	3117	0.031
	2	67.3	228548	3068	0.031
	3	66.8	222482	2967	0.031
	4	65.9	217166	2911	0.032
	5	65.9	223025	3000	0.032
	6	65.9	224845	2912	0.030
Average for Sample 9		66.60299	228216.7	2995.842	0.0312339
Standard Dev for Sample 9		0.89421	12800.89	83.85731	0.000677
Coefficient of Variation for Sample 9		1.34%	5.61%	2.80%	2.17%
**14**	1	68.7	281885	3516	0.032
	2	68.3	284365	3617	0.036
	3	69.5	287167	3630	0.040
	4	68.4	276612	3525	0.037
	5	69.0	283960	3630	0.040
	6	67.8	254077	3062	0.029
Average for Sample 9		68.61325	278011	3496.669	0.035601
Standard Dev for Sample 9		0.586232	12242.43	219.3349	0.0044951
Coefficient of Variation for Sample 9		0.85%	4.40%	6.27%	12.63%
**15**	1	71.3	320421	3605	0.029
	2	70.8	301256	3362	0.028
	3	69.1	271862	3213	0.025
	4	70.8	285917	3429	0.030
	5	71.7	284026	3197	0.024
	6	71.1	276940	3261	0.028
Average for Sample 9		70.79461	290070.3	3344.423	0.0272626
Standard Dev for Sample 9		0.916791	17910.97	155.7952	0.0022778
Coefficient of Variation for Sample 9		1.30%	6.17%	4.66%	8.35%
**16**	1	74.7	315250	3217	0.020
	2	74.3	318137	3200	0.018
	3	73.8	318249	3193	0.021
	4	73.8	333512	3362	0.020
	5	74.3	325278	3312	0.020
	6	73.9	351404	3509	0.020
Average for Sample 9		74.12573	326971.8	3298.872	0.0195533
Standard Dev for Sample 9		0.342014	13668.4	123.3658	0.0009529
Coefficient of Variation for Sample 9		0.46%	4.18%	3.74%	4.87%
**17**	1	68.3	313737	4687	0.029
	2	68.7	315917	4699	0.029
	3	68.6	318432	4675	0.030
	4	67.9	308634	4544	0.032
	5	69.8	337378	4865	0.028
	6	68.2	342063	4638	0.024
Average for Sample 9		68.57234	322693.4	4684.823	0.0286881
Standard Dev for Sample 9		0.661847	13658.72	104.6904	0.002599
Coefficient of Variation for Sample 9		0.97%	4.23%	2.23%	9.06%
**18**	1	71.5	370286	4853	0.023
	2	72.3	369850	4740	0.021
	3	71.8	356905	4819	0.024
	4	71.7	370702	4935	0.024
	5	72.6	395546	5080	0.023
	6	71.3	362564	4816	0.024
Average for Sample 9		71.8598	370975.6	4873.862	0.0229549
Standard Dev for Sample 9		0.507577	13223.09	119.2369	0.0010593
Coefficient of Variation for Sample 9		0.71%	3.56%	2.45%	4.61%
**19**	1	75.2	442821	5050	0.017
	2	75.3	452187	5361	0.019
	3	74.3	452300	5362	0.018
	4	74.7	449912	5472	0.019
	5	74.5	447304	5209	0.017
	6	73.9	428241	5045	0.018
Average for Sample 9		74.62356	445460.9	5250.078	0.0180697
Standard Dev for Sample 9		0.549032	9151.374	177.7501	0.0009402
Coefficient of Variation for Sample 9		0.74%	2.05%	3.39%	5.20%
**20**	1	63.7	185425	2747	0.040
	2	63.8	194336	2798	0.038
	3	63.3	193889	2747	0.035
	4	62.9	187341	2751	0.039
	5	62.8	198313	2906	0.040
	6	63.5	176282	2581	0.036
Average for Sample 9		63.3409	189264.3	2755.039	0.0379585
Standard Dev for Sample 9		0.398893	7949.511	104.971	0.001967
Coefficient of Variation for Sample 9		0.63%	4.20%	3.81%	5.18%
**21**	1	67.6	264866	3054	0.026
	2	69.1	289177	3143	0.021
	3	70.1	298515	3238	0.021
	4	69.4	287939	3128	0.021
	5	69.6	289445	3132	0.022
	6	69.2	283538	3157	0.023
Average for Sample 9		69.15271	285580	3142.15	0.0223883
Standard Dev for Sample 9		0.8401	11260.37	59.25396	0.0016925
Coefficient of Variation for Sample 9		1.21%	3.94%	1.89%	7.56%
**22**	1	73.8	341346	3299	0.015
	2	74.5	356025	3411	0.014
	3	73.4	345361	3258	0.015
	4	74.6	361624	3255	0.014
	5	74.5	356084	3329	0.015
	6	73.4	401193	3233	0.015
Average for Sample 22		74.01528	360272.1	3297.656	0.0147444
Standard Dev for Sample 22		0.566825	21412.51	65.46746	0.0006242
Coefficient of Variation for Sample 22		0.77%	5.94%	1.99%	4.23%
**23**	1	71.4	282006	3531	0.027
	2	71.3	304162	3490	0.027
	3	71.9	304084	3465	0.025
	4	71.7	293589	3435	0.027
	5	71.7	310138	3484	0.023
	6	71.9	309004	3610	0.034
Average for Sample 22		71.7	300497	3503	0.027
Standard Dev for Sample 22		0.3	10782	61	0.003
Coefficient of Variation for Sample 22		0.4%	3.6%	1.7%	12.8%
**24**	1	70.1	300777	3453	0.024
	2	70.4	306160	3498	0.025
	3	70.5	312452	3618	0.027
	4	70.1	293275	3452	0.029
	5	70.4	316206	3560	0.032
	6	70.2	291270	3418	0.028
Average for Sample 22		70.3	303357	3500	0.027
Standard Dev for Sample 22		0.2	10100	76	0.003
Coefficient of Variation for Sample 22		0.2%	3.3%	2.2%	9.9%
**25**	1	68.3	283715	3432	0.029
	2	68.6	286436	3490	0.033
	3	68.6	270289	3245	0.030
	4	68.5	279594	3291	0.028
	5	68.7	267705	3284	0.032
	6	67.8	290246	3506	0.032
Average for Sample 22		68.4	279664	3375	0.031
Standard Dev for Sample 22		0.3	9002	115	0.002
Coefficient of Variation for Sample 22		0.5%	3.2%	3.4%	6.9%
**26**	1	77.4	545238	6419	0.016
	2	77.2	544048	6582	0.017
	3	76.8	532361	6751	0.019
	4	76.8	545090	6688	0.018
	5	77.0	532565	6204	0.016
	6	76.6	529209	6758	0.019
Average for Sample 22		77.0	538085	6567	0.017
Standard Dev for Sample 22		0.3	7454	219	0.001
Coefficient of Variation for Sample 22		0.4%	1.4%	3.3%	7.8%
**27**	1	80.0	620059	6886	0.015
	2	80.5	673533	7054	0.015
	3	81.1	661816	7092	0.015
	4	80.9	665518	7046	0.015
	5	81.2	691196	7058	0.015
	6	80.2	640541	6799	0.015
Average for Sample 22		80.6	658777	6989	0.015
Standard Dev for Sample 22		0.5	25120	118	0.000
Coefficient of Variation for Sample 22		0.6%	3.8%	1.7%	2.5%
**28**	1	77.4	361130	3203	0.010
	2	76.7	376812	3409	0.011
	3	77.6	376196	3372	0.012
	4	77.4	365807	3398	0.013
	5	77.4	381155	3434	0.011
	6	77.8	398580	3603	0.013
Average for Sample 22		77.4	376613	3403	0.012
Standard Dev for Sample 22		0.4	13114	128	0.001
Coefficient of Variation for Sample 22		0.5%	3.5%	3.8%	11.1%
**29**	1	79.2	370178	3102	0.011
	2	79.0	328677	2958	0.013
	3	80.1	362286	3231	0.011
	4	78.4	317604	3034	0.013
	5	79.5	361432	3210	0.011
	6	76.9	339470	3015	0.012
Average for Sample 22		78.8	346608	3092	0.012
Standard Dev for Sample 22		1.1	21142	110	0.001
Coefficient of Variation for Sample 22		1.4%	6.1%	3.6%	8.0%

The coefficient of linear thermal expansion (CLTE often referred to as “α”) is a material property which characterizes the ability of a plastic to expand under the effect of temperature elevation. For obtaining coefficient of linear thermal expansion (CLTE) of extruded fiber-reinforced composites manufactured from recycled wind turbine blade (rWTB) material, tests were performed based on ASTM D1037-12 [2]. Presented dataset include influence of mill screen size (MSS), resin content and coupling agent [3] on the CLTE of second-generation composites (Table 4, Table 5).

**Table 4:** CLTE data (first cycle) of extruded fiber-reinforced composites manufactured from recycled wind turbine blade material.

Sample	Freezer (inch)	Room Temp (inch)	60C (inch)	Freezer (cm)	Room Temp (cm)	60C (cm)	CLTE results	Ave × E−5	STD × E−06	COV
1–1	11.899	11.909	11.923	30.22346	30.24886	30.28442	2.1439E-05	3.0729	5.3046	0.172623
1–2	11.895	11.913	11.933	30.2133	30.25902	30.30982	3.3934E-05			
1–3	11.875	11.899	11.91	30.1625	30.22346	30.2514	3.1292E-05			
1–4	11.888	11.909	11.926	30.19552	30.24886	30.29204	3.3945E-05			
1–5	11.885	11.915	11.922	30.1879	30.2641	30.28188	3.3035E-05			
2–1	11.891	11.912	11.928	30.20314	30.25648	30.29712	3.3044E-05	3.2517	3.8858	0.1195
2–2	11.884	11.903	11.925	30.18536	30.23362	30.2895	3.6644E-05			
2–3	11.886	11.906	11.926	30.19044	30.24124	30.29204	3.5741E-05			
2–4	11.885	11.905	11.918	30.1879	30.2387	30.27172	2.9489E-05			
2–5	11.891	11.92	11.922	30.20314	30.2768	30.28188	2.7667E-05			
3–1	11.895	11.913	11.926	30.2133	30.25902	30.29204	2.7683E-05	3.2152	3.2193	0.100127
3–2	11.886	11.911	11.927	30.19044	30.25394	30.29458	3.6619E-05			
3–3	11.89	11.919	11.927	30.2006	30.27426	30.29458	3.3024E-05			
3–4	11.885	11.905	11.92	30.1879	30.2387	30.2768	3.1276E-05			
3–5	11.889	11.909	11.925	30.19806	30.24886	30.2895	3.2159E-05			
4–1	11.872	11.906	11.922	30.15488	30.24124	30.28188	4.4676E-05	3.8758	4.407	0.113715
4–2	11.879	11.904	11.924	30.17266	30.23616	30.28696	4.0215E-05			
4–3	11.891	11.914	11.93	30.20314	30.26156	30.3022	3.4824E-05			
4–4	11.899	11.929	11.944	30.22346	30.29966	30.33776	4.0131E-05			
4–5	11.886	11.91	11.924	30.19044	30.2514	30.28696	3.3943E-05			
5–1	11.875	11.907	11.925	30.1625	30.24378	30.2895	4.4672E-05	3.8229	5.4812	0.143377
5–2	11.883	11.906	11.918	30.18282	30.24124	30.27172	3.1273E-05			
5–3	11.887	11.915	11.935	30.19298	30.2641	30.3149	4.2857E-05			
5–4	11.89	11.909	11.93	30.2006	30.24886	30.3022	3.5732E-05			
5–5	11.89	11.914	11.931	30.2006	30.26156	30.30474	3.661E-05			
6–1	11.887	11.911	11.927	30.19298	30.25394	30.29458	3.5726E-05	3.6988	3.1457	0.085045
6–2	11.883	11.908	11.926	30.18282	30.24632	30.29204	3.8415E-05			
6–3	11.89	11.91	11.926	30.2006	30.2514	30.29204	3.2156E-05			
6–4	11.886	11.913	11.929	30.19044	30.25902	30.29966	3.8399E-05			
6–5	11.871	11.895	11.916	30.15234	30.2133	30.26664	4.0246E-05			
7–1	11.883	11.907	11.923	30.18282	30.24378	30.28442	3.5738E-05	3.252E	2.868	0.088193
7–2	11.89	11.911	11.929	30.2006	30.25394	30.29966	3.4833E-05			
7–3	11.883	11.904	11.918	30.18282	30.23616	30.27172	3.1279E-05			
7–4	11.882	11.904	11.918	30.18028	30.23616	30.27172	3.2172E-05			
7–5	11.893	11.912	11.925	30.20822	30.25648	30.2895	2.8578E-05			
8–1	11.89	11.901	11.911	30.2006	30.22854	30.25394	3.0385E-05	3.1282	5.0106	0.160172
8–2	11.888	11.904	11.922	30.19552	30.23616	30.28188	3.0385E-05			
8–3	11.876	11.893	11.909	30.16504	30.20822	30.24886	2.9519E-05			
8–4	11.895	11.914	11.925	30.2133	30.26156	30.2895	2.6788E-05			
8–5	11.882	11.901	11.925	30.18028	30.22854	30.2895	3.8438E-05			
9–1	11.887	11.903	11.92	30.19298	30.23362	30.2768	2.9494E-05	2.9856	2.8661	0.095998
9–2	11.884	11.897	11.915	30.18536	30.21838	30.2641	2.772E-05			
9–3	11.882	11.9	11.92	30.18028	30.226	30.2768	3.3971E-05			
9–4	11.889	11.906	11.924	30.19806	30.24124	30.28696	3.1273E-05			
9–5	11.885	11.9	11.915	30.1879	30.226	30.2641	2.6819E-05			
10–1	11.864	11.892	11.914	30.13456	30.20568	30.26156	4.4729E-05	4.3621	2.4979	0.057263
10–2	11.874	11.905	11.921	30.15996	30.2387	30.27934	4.1999E-05			
10–3	11.87	11.904	11.92	30.1498	30.23616	30.2768	4.4684E-05			
10–4	11.882	11.909	11.927	30.18028	30.24886	30.29458	4.0198E-05			
10–5	11.869	11.898	11.921	30.14726	30.22092	30.27934	4.6494E-05			
11–1	11.878	11.903	11.925	30.17012	30.23362	30.2895	4.2006E-05	4.3647	5.5797	0.127838
11–2	11.876	11.892	11.919	30.16504	30.20568	30.27426	3.8467E-05			
11–3	11.88	11.902	11.938	30.1752	30.23108	30.32252	5.1842E-05			
11–4	11.836	11.864	11.888	30.06344	30.13456	30.19552	4.6628E-05			
11–5	11.889	11.913	11.933	30.19806	30.25902	30.30982	3.9292E-05			
12–1	11.88	11.904	11.922	30.1752	30.23616	30.28188	3.7534E-05	3.5373	5.4022	0.152721
12–2	11.879	11.899	11.923	30.17266	30.22346	30.28442	3.9338E-05			
12–3	11.9	11.923	11.929	30.226	30.28442	30.29966	2.5875E-05			
12–4	11.882	11.904	11.924	30.18028	30.23616	30.28696	3.7534E-05			
12–5	11.899	11.923	11.94	30.22346	30.28442	30.3276	3.6582E-05			
13–1	11.886	11.911	11.93	30.19044	30.25394	30.3022	3.9299E-05	4.2172	6.1732	0.146382
13–2	11.874	11.903	11.927	30.15996	30.23362	30.29458	4.7369E-05			
13–3	11.88	11.905	11.923	30.1752	30.2387	30.28442	3.8425E-05			
13–4	11.873	11.908	11.929	30.15742	30.24632	30.29966	5.0029E-05			
13–5	11.878	11.907	11.918	30.17012	30.24378	30.27172	3.5738E-05			
14–1	11.888	11.915	11.934	30.19552	30.2641	30.31236	4.1071E-05	3.3407	5.2293	0.156532
14–2	11.884	11.907	11.922	30.18536	30.24378	30.28188	3.3951E-05			
14–3	11.876	11.902	11.915	30.16504	30.23108	30.2641	3.4859E-05			
14–4	11.888	11.914	11.921	30.19552	30.26156	30.27934	2.9466E-05			
14–5	11.89	11.911	11.921	30.2006	30.25394	30.27934	2.7688E-05			
15–1	11.875	11.899	11.917	30.1625	30.22346	30.26918	3.755E-05	3.198E	4.072E	0.12733
15–2	11.895	11.92	11.932	30.2133	30.2768	30.30728	3.3022E-05			
15–3	11.896	11.911	11.926	30.21584	30.25394	30.29204	2.6794E-05			
15–4	11.882	11.904	11.915	30.18028	30.23616	30.2641	2.9491E-05			
15–5	11.89	11.912	11.927	30.2006	30.25648	30.29458	3.3044E-05			
16–1	11.868	11.897	11.925	30.14472	30.21838	30.2895	5.0969E-05	4.5967	3.4899	0.075921
16–2	11.868	11.89	11.914	30.14472	30.2006	30.26156	4.1157E-05			
16–3	11.876	11.903	11.927	30.16504	30.23362	30.29458	4.5581E-05			
16–4	11.866	11.893	11.917	30.13964	30.20822	30.26918	4.562E-05			
16–5	11.865	11.895	11.917	30.1371	30.2133	30.26918	4.6506E-05			
17–1	11.869	11.89	11.911	30.14726	30.2006	30.25394	3.7579E-05	4.2203	2.769E	0.06561
17–2	11.873	11.897	11.92	30.15742	30.21838	30.2768	4.2027E-05			
17–3	11.877	11.908	11.926	30.16758	30.24632	30.29204	4.3775E-05			
17–4	11.873	11.899	11.923	30.15742	30.22346	30.28442	4.4702E-05			
17–5	11.868	11.894	11.916	30.14472	30.21076	30.26664	4.2932E-05			
18–1	11.878	11.898	11.917	30.17012	30.22092	30.26918	3.4871E-05	3.7196	2.9488	0.079277
18–2	11.884	11.906	11.922	30.18536	30.24124	30.28188	3.3954E-05			
18–3	11.87	11.891	11.915	30.1498	30.20314	30.2641	4.0259E-05			
18–4	11.874	11.9	11.919	30.15996	30.226	30.27426	4.0229E-05			
18–5	11.88	11.896	11.921	30.1752	30.21584	30.27934	3.6665E-05			
19–1	11.869	11.894	11.906	30.14726	30.21076	30.24124	3.3094E-05	3.6216	6.5554	0.18101
19–2	11.878	11.9	11.91	30.17012	30.226	30.2514	2.8607E-05			
19–3	11.87	11.891	11.918	30.1498	30.20314	30.27172	4.2943E-05			
19–4	11.879	11.903	11.924	30.17266	30.23362	30.28696	4.0219E-05			
19–5	11.7	11.893	11.902	29.718	30.20822	30.23108	4.0219E-05			
20–1	11.882	11.906	11.919	30.18028	30.24124	30.27426	3.306E-05	2.7537	3.4192	0.124166
20–2	11.877	11.896	11.909	30.16758	30.21584	30.24886	2.8617E-05			
20–3	11.896	11.907	11.924	30.21584	30.24378	30.28696	2.5017E-05			
20–4	11.873	11.892	11.902	30.15742	30.20568	30.23108	2.5943E-05			
20–5	11.877	11.892	11.905	30.16758	30.20568	30.2387	2.5048E-05			
21–1	11.884	11.9	11.916	30.18536	30.226	30.26664	2.8607E-05	2.9689	3.0797	0.103734
21–2	11.866	11.889	11.901	30.13964	30.19806	30.22854	3.1318E-05			
21–3	11.881	11.89	11.918	30.17774	30.2006	30.27172	3.3105E-05			
21–4	11.874	11.895	11.908	30.15996	30.2133	30.24632	3.0408E-05			
21–5	11.89	11.912	11.918	30.2006	30.25648	30.27172	2.5006E-05			
22–1	11.873	11.897	11.907	30.15742	30.21838	30.24378	3.0403E-05	3.2365	5.8128	0.179602
22–2	11.885	11.903	11.911	30.1879	30.23362	30.25394	2.3237E-05			
22–3	11.881	11.903	11.922	30.17774	30.23362	30.28188	3.6644E-05			
22–4	11.873	11.892	11.915	30.15742	30.20568	30.2641	3.7572E-05			
22–5	11.878	11.901	11.916	30.17012	30.22854	30.26664	3.3968E-05

**Table 5:** CLTE data (second cycle) of extruded fiber-reinforced composites manufactured from recycled wind turbine blade material.

Sample	Freezer (inch)	Room Temp (inch)	60C (inch)	Freezer (cm)	Room Temp (cm)	60C (cm)	CLTE results	Ave × E−5	STD × E−06	COV
1–1	11.885	11.902	11.921	30.1879	30.23108	30.27934	3.2178E-05	3.79	1.21	0.31838
1–2	11.89	11.914	11.925	30.2006	30.26156	30.2895	3.1252E-05			
1–3	11.861	11.89	11.927	30.12694	30.2006	30.29458	5.9052E-05			
1–4	11.885	11.906	11.926	30.1879	30.24124	30.29204	3.6634E-05			
1–5	11.886	11.904	11.92	30.19044	30.23616	30.2768	3.0385E-05			
2–1	11.885	11.908	11.929	30.1879	30.24632	30.29966	3.9308E-05	3.36	5.84	0.173848
2–2	11.886	11.909	11.93	30.19044	30.24886	30.3022	3.9305E-05			
2–3	11.884	11.907	11.916	30.18536	30.24378	30.26664	2.859E-05			
2–4	11.885	11.903	11.915	30.1879	30.23362	30.2641	2.6812E-05			
2–5	11.886	11.909	11.924	30.19044	30.24886	30.28696	3.3945E-05			
3–1	11.887	11.91	11.929	30.19298	30.2514	30.29966	3.7515E-05	3.16	6.75	0.213569
3–2	11.886	11.915	11.927	30.19044	30.2641	30.29458	3.6607E-05			
3–3	11.894	11.911	11.929	30.21076	30.25394	30.29966	3.126E-05			
3–4	11.894	11.902	11.917	30.21076	30.23108	30.26918	2.0558E-05			
3–5	11.887	11.909	11.923	30.19298	30.24886	30.28442	3.2159E-05			
4–1	11.868	11.891	11.918	30.14472	30.20314	30.27172	4.4733E-05	4.29	3.28	0.076546
4–2	11.873	11.901	11.923	30.15742	30.22854	30.28442	4.4695E-05			
4–3	11.881	11.909	11.928	30.17774	30.24886	30.29712	4.1985E-05			
4–4	11.893	11.924	11.944	30.20822	30.28696	30.33776	4.5501E-05			
4–5	11.881	11.908	11.923	30.17774	30.24632	30.28442	3.7522E-05			
5–1	11.872	11.904	11.93	30.15488	30.23616	30.3022	5.1833E-05	4.02	7.38	0.183436
5–2	11.881	11.903	11.92	30.17774	30.23362	30.2768	3.4856E-05			
5–3	11.889	11.914	11.931	30.19806	30.26156	30.30474	3.7503E-05			
5–4	11.883	11.901	11.931	30.18282	30.22854	30.30474	4.2907E-05			
5–5	11.889	11.909	11.927	30.19806	30.24886	30.29458	3.3945E-05			
6–1	11.879	11.908	11.926	30.17266	30.24632	30.29204	4.1989E-05	3.63	3.6	0.09937
6–2	11.885	11.91	11.924	30.1879	30.2514	30.28696	3.4836E-05			
6–3	11.885	11.908	11.927	30.1879	30.24632	30.29458	3.7522E-05			
6–4	11.889	11.91	11.926	30.19806	30.2514	30.29204	3.3049E-05			
6–5	11.875	11.899	11.913	30.1625	30.22346	30.25902	3.3974E-05			
7–1	11.882	11.906	11.921	30.18028	30.24124	30.27934	3.4847E-05	3.36	3.26	0.097156
7–2	11.888	11.915	11.928	30.19552	30.2641	30.29712	3.5714E-05			
7–3	11.883	11.903	11.924	30.18282	30.23362	30.28696	3.6644E-05			
7–4	11.885	11.905	11.921	30.1879	30.2387	30.27934	3.217E-05			
7–5	11.888	11.914	11.92	30.19552	30.26156	30.2768	2.8574E-05			
8–1	11.891	11.909	11.917	30.20314	30.24886	30.26918	2.3226E-05	2.45	1.49	0.060788
8–2	11.89	11.91	11.92	30.2006	30.2514	30.2768	2.6797E-05			
8–3	11.881	11.898	11.908	30.17774	30.22092	30.24632	2.4141E-05			
8–4	11.895	11.921	11.923	30.2133	30.27934	30.28442	2.4987E-05			
8–5	11.887	11.903	11.913	30.19298	30.23362	30.25902	2.3237E-05			
9–1	11.885	11.902	11.924	30.1879	30.23108	30.28696	3.4859E-05	3.02	5.34	0.176771
9–2	11.886	11.903	11.915	30.19044	30.23362	30.2641	2.5919E-05			
9–3	11.882	11.91	11.921	30.18028	30.2514	30.27934	3.4836E-05			
9–4	11.89	11.915	11.926	30.2006	30.2641	30.29204	3.2143E-05			
9–5	11.893	11.91	11.919	30.20822	30.2514	30.27426	2.3224E-05			
10–1	11.866	11.89	11.916	30.13964	30.2006	30.26664	4.4736E-05	3.51	7.83	0.222906
10–2	11.864	11.899	11.923	30.13456	30.22346	30.28442	5.2749E-05			
10–3	11.865	11.893	11.911	30.1371	30.20822	30.25394	4.1147E-05			
10–4	11.87	11.896	11.921	30.1498	30.21584	30.27934	4.5608E-05			
10–5	11.876	11.893	11.911	30.16504	30.20822	30.25394	3.1308E-05			
11–1	11.875	11.902	11.919	30.1625	30.23108	30.27426	3.9328E-05	3.59	4.67	0.129839
11–2	11.874	11.898	11.909	30.15996	30.22092	30.24886	3.1294E-05			
11–3	11.888	11.908	11.933	30.19552	30.24632	30.30982	4.0202E-05			
11–4	11.844	11.868	11.878	30.08376	30.14472	30.17012	3.0477E-05			
11–5	11.895	11.914	11.938	30.2133	30.26156	30.32252	3.8396E-05			
12–1	11.88	11.901	11.934	30.1752	30.22854	30.31236	4.8271E-05	3.58	5.18	0.144575
12–2	11.883	11.903	11.937	30.18282	30.23362	30.31998	4.8262E-05			
12–3	11.893	11.919	11.935	30.20822	30.27426	30.3149	3.7487E-05			
12–4	11.886	11.912	11.934	30.19044	30.25648	30.31236	4.2868E-05			
12–5	11.898	11.921	11.941	30.22092	30.27934	30.33014	3.8373E-05			
13–1	11.872	11.905	11.929	30.15488	30.2387	30.29966	5.0935E-05	5.11	6.74	0.131787
13–2	11.869	11.897	11.922	30.14726	30.21838	30.28188	4.7393E-05			
13–3	11.871	11.908	11.935	30.15234	30.24632	30.3149	5.7176E-05			
13–4	11.866	11.905	11.931	30.13964	30.2387	30.30474	5.8084E-05			
13–5	11.87	11.894	11.917	30.1498	30.21076	30.26918	4.2038E-05			
14–1	11.885	11.915	11.925	30.1879	30.2641	30.2895	3.5714E-05	4	4.46	0.111285
14–2	11.877	11.904	11.924	30.16758	30.23616	30.28696	4.2003E-05			
14–3	11.872	11.899	11.922	30.15488	30.22346	30.28188	4.4702E-05			
14–4	11.873	11.897	11.921	30.15742	30.21838	30.27934	4.2922E-05			
14–5	11.881	11.901	11.92	30.17774	30.22854	30.2768	3.4862E-05			
15–1	11.875	11.9	11.921	30.1625	30.226	30.27934	4.1123E-05	3.75	4.86	0.129489
15–2	11.892	11.913	11.933	30.20568	30.25902	30.30982	3.6613E-05			
15–3	11.886	11.916	11.932	30.19044	30.26664	30.30728	4.1068E-05			
15–4	11.881	11.909	11.925	30.17774	30.24886	30.2895	3.9305E-05			
15–5	11.895	11.913	11.928	30.2133	30.25902	30.29712	2.9469E-05			
16–1	11.867	11.892	11.921	30.14218	30.20568	30.27934	4.8307E-05	4.24	3.56	0.083979
16–2	11.867	11.894	11.913	30.14218	30.21076	30.25902	4.1144E-05			
16–3	11.874	11.89	11.922	30.15996	30.2006	30.28188	4.2947E-05			
16–4	11.869	11.894	11.914	30.14726	30.21076	30.26156	4.0249E-05			
16–5	11.871	11.896	11.915	30.15234	30.21584	30.2641	3.9348E-05			
17–1	11.871	11.894	11.91	30.15234	30.21076	30.2514	3.4883E-05	3.9	6.16	0.158173
17–2	11.876	11.901	11.921	30.16504	30.22854	30.27934	4.0225E-05			
17–3	11.88	11.907	11.935	30.1752	30.24378	30.3149	4.914E-05			
17–4	11.879	11.906	11.92	30.17266	30.24124	30.2768	3.6634E-05			
17–5	11.873	11.895	11.911	30.15742	30.2133	30.25394	3.3985E-05			
18–1	11.879	11.901	11.914	30.17266	30.22854	30.26156	3.1286E-05	3.29	1.47	0.044819
18–2	11.884	11.907	11.922	30.18536	30.24378	30.28188	3.3951E-05			
18–3	11.871	11.894	11.909	30.15234	30.21076	30.24886	3.3988E-05			
18–4	11.881	11.907	11.916	30.17774	30.24378	30.26664	3.1271E-05			
18–5	11.879	11.9	11.917	30.17266	30.226	30.26918	3.3971E-05			
19–1	11.871	11.888	11.901	30.15234	30.19552	30.22854	2.6846E-05	3.33	4.78	0.143792
19–2	11.874	11.896	11.916	30.15996	30.21584	30.26664	3.756E-05			
19–3	11.87	11.898	11.913	30.1498	30.22092	30.25902	3.8447E-05			
19–4	11.881	11.907	11.917	30.17774	30.24378	30.26918	3.2164E-05			
19–5	11.872	11.891	11.907	30.15488	30.20314	30.24378	3.1313E-05			
20–1	11.88	11.899	11.914	30.1752	30.22346	30.26156	3.0398E-05	2.74	3.06	0.111958
20–2	11.875	11.895	11.908	30.1625	30.2133	30.24632	2.9514E-05			
20–3	11.889	11.912	11.921	30.19806	30.25648	30.27934	2.8578E-05			
20–4	11.871	11.893	11.897	30.15234	30.20822	30.21838	2.3257E-05			
20–5	11.881	11.892	11.909	30.17774	30.20568	30.24886	2.5048E-05			
21–1	11.88	11.899	11.912	30.1752	30.22346	30.25648	2.861E-05	2.65	1.62	#DIV/0!
21–2	11.871	11.889	11.899	30.15234	30.19806	30.22346	2.5054E-05			
21–3	11.881	11.903	11.909	30.17774	30.23362	30.24886	2.5025E-05			
21–4	11.875	11.906	11.906	30.1625	30.24124	30.24124	2.7699E-05			
21–5	11.889	11.912	11.918	30.19806	30.25648	30.27172	2.5899E-05			
22–1	11.876	11.902	11.908	30.16504	30.23108	30.24632	2.8602E-05	2.72	3.39	0.12472
22–2	11.88	11.901	11.906	30.1752	30.22854	30.24124	2.3241E-05			
22–3	11.885	11.908	11.917	30.1879	30.24632	30.26918	2.8588E-05			
22–4	11.873	11.893	11.908	30.15742	30.20822	30.24632	3.1308E-05			
22–5	11.882	11.91	11.909	30.18028	30.2514	30.24886	2.4117E-05			

## 2 Experimental design, materials, and methods

### 2.1 Materials

Recycled wind turbine blade (rWTB) material was supplied by Global Fiberglass Solutions at an incoming moisture content of 1.25% and shipped to the Composites Materials and Engineering Center at Washington State University. A high-density polyethylene (HDPE) (0.3 MFI) was obtained from a commercial vender and used as the matrix for the second-generation extruded composite. The rWTB material was hammer-milled through 3.18, and 1.59 mm screen size (MSS) and particle size distribution of the refined material was performed with Ro-Tap sieve analysis procedures [4]. A commercially available 60-mesh pine (P. stobus) was used for baseline comparison to the rWTB filled extrudate. Methacryloxypropyltrimethoxysilane (Silane) (Gelest Inc.) and maleic anhydride polyethylene (MAPE) were used as the coupling agents [3].

### 2.2 Extruded rWTB composite preparation

The various milled size fractions of rWTB material were mixed with high density polyethylene, non-metallic stearate lubricant, MAPE and silane as coupling agents were also added to the formulation. Silane was received in a liquid form and sprayed to rWTB materials. They were then blended for 15 minutes and dried for 10 hours at 60 °C in an oven [5]. MAPE was added to the dry blend as a pellet. For comparison purposes, a commercial 60-mesh pine was used as a feedstock source. Mechanical tests were performed based on ASTM D790-17 [6], [7].

### 2.3 Mechanical properties of extruded rWTB composites

The mechanical properties of the extruded composites were obtained from flexural tests. To evaluate the influence of MSS on the mechanical properties, milled material from 3.18 to 1.59 mm MSS was chosen. data show that decreasing MSS decreased both modulus of elasticity (MOE) and modulus of rupture (MOR) while the strain at break (SB) remained consistent as shown in Table 1. While MSS had a slightly significant influence on the MOE, it did not have a significant influence on MOR and SB. When the level of rWTB was changed, all of the mechanical properties varied significantly as well. Addition of more rWTB to the mix increased the MOE and lowered the SB, while the MOR remained constant. Table 2.

MAPE and silane are two common coupling agents that are used in the production of reinforced thermoplastic composites. Table 3 presents the mechanical properties of composites modified with MAPE and silane. As expected, the results indicate that MAPE had a significantly positive influence on MOE and MOR of the composite. When the rWTB content increased, the MAPE had a slightly stronger influence on the MOE and MOR of the composite. The silane-based coupling agent showed limited improvement, and in some cases a reduction in the mechanical properties of composites at the lower rWTB levels. When the rWTB content increased, the influence of silane on MOE and MOR decreased. However, this effect was not significant. In addition, both the MAPE and silane reduced the SB of the composite. Because silane lacked performance, it was not used at higher level of rWTB content. As the rWTB level was increased to over 65%, there was a separation in behavior for the MAPE based composites. Results show that for the non-MAPE composites, increasing the rWTB content more than 65% decreased the MOE and MOR. However, MAPE had a significant influence on the MOE and MOR of composites with a higher rWTB content. By increasing rWTB content to more than 65%, the difference in MOE and MOR between untreated composite and modified with MAPE composite was significant. For a 70% rWTB content, the MOE and MOR of the composite modified with MAPE was almost twice that of the untreated composite. The decreasing trend of SB with increasing rWTB showed a leveling-off or plateau after 65%.

### 2.4 Coefficient of linear thermal expansion (CLTE)

Table 4, Table 5 presents CLTE data (two cycles). Data shows that increasing rWTB content decreased CLTE for both untreated composite and modified with MAPE composite. The addition of MAPE did not alter the behavior of the CLTE. CLTE data for rWTB blending with pine show that by increasing the rWTB content from 0% to 75%, CLTE increased. However, when this increased to 100%, CLTE started to decrease. Data indicates that MSS had no significant influence on CLTE of composites, and that simply decreasing MSS increased CLTE slightly [1].
